# Supplementary figures and images for: Phylogenetic Species of Paracoccidioides spp. Isolated from Clinical and Environmental Samples in a Hyperendemic Area of Paracoccidioidomycosis in Southeastern Brazil
Source: J Fungi (Basel). 2020 Aug 11;6(3):132. doi: 10.3390/jof6030132 (PMC7559761; doi:10.3390/jof6030132)

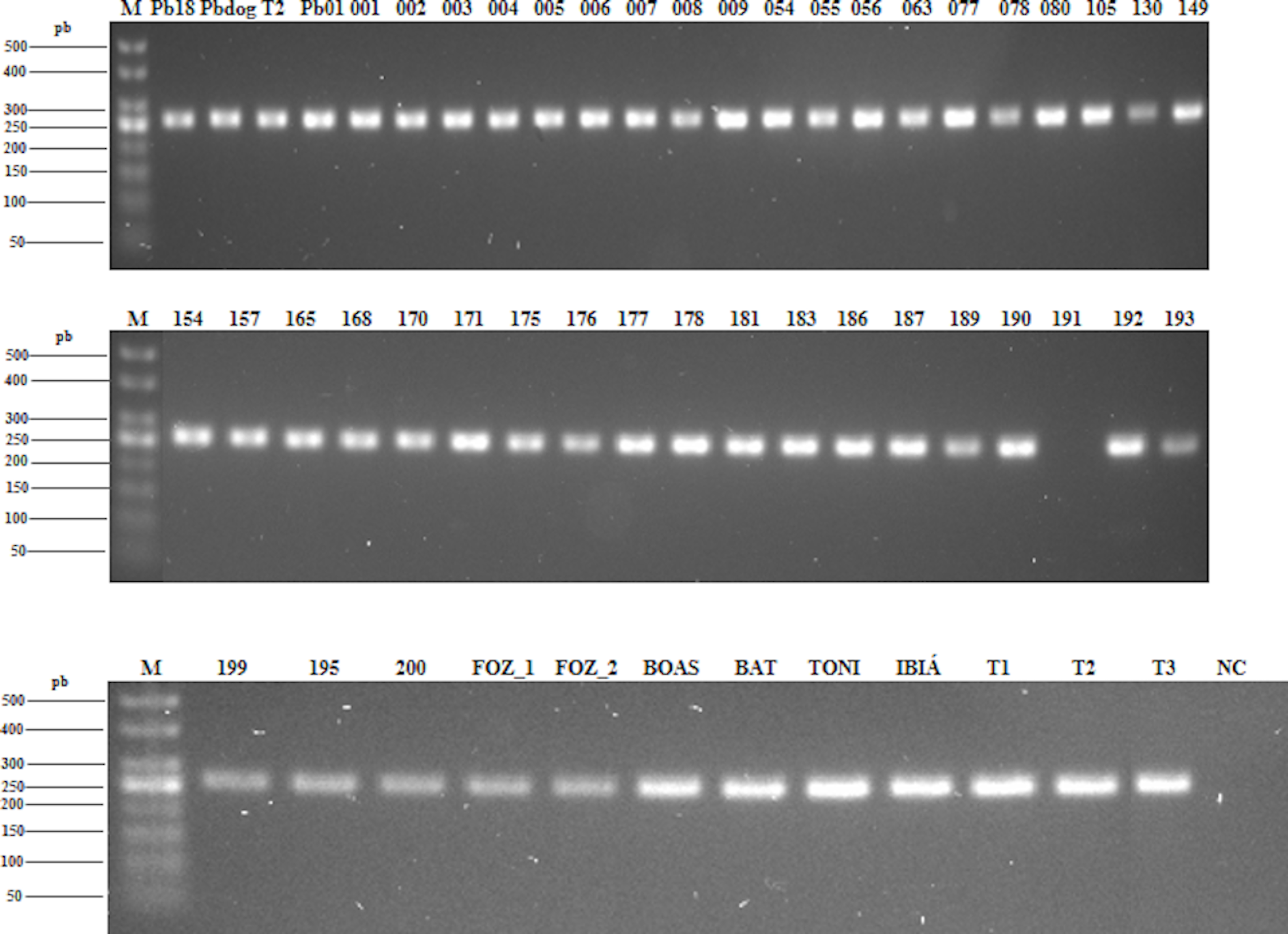

Supplement: Supplementary file 1 [file jof-06-00132-s001.zip › Figure S1.tif]
