# Supplementary material for: Phylogenetic Species of Paracoccidioides spp. Isolated from Clinical and Environmental Samples in a Hyperendemic Area of Paracoccidioidomycosis in Southeastern Brazil
Source: J Fungi (Basel). 2020 Aug 11;6(3):132. doi: 10.3390/jof6030132 (PMC7559761; doi:10.3390/jof6030132)
Supplement: Supplementary file 1 [file jof-06-00132-s001.zip › Article_Tiago Cocio_TableS1_22072020.doc]

**Table S1.** Genotypic characterization of clinical isolates of *Paracoccidioides* spp.: source, clinical PCM form, residence, identification method, and GenBank.

|  |  |  | | ***Identification method*** | |  |
| --- | --- | --- | --- | --- | --- | --- |
| ***Isolate*** | ***Source*** | ***Clinical Form*** | ***Residence*** | ***PCR-RFLP***  ***tub1*** | ***gp43 exon 2 sequencing*** | ***GenBank*** |
| HCRP 001 | Lymph node | Acute | Monte Santo de Minas – MG | *P. brasiliensis* *sensu stricto* S1 | *P. brasiliensis sensu stricto* S1b | MK909758 |
| HCRP 002 | Lymph node | Chronic | Ribeirão Preto – SP | *P. brasiliensis* *sensu stricto* S1 | *P. brasiliensis sensu stricto* S1b | MK909759 |
| HCRP 003 | Lymph node | Subacute | Guaíra – SP | *P. brasiliensis* *sensu stricto* S1 | *P. brasiliensis sensu stricto* S1b | MK909760 |
| HCRP 004 | Sputum | Chronic | Serrana – SP | *P. brasiliensis* *sensu stricto* S1 | *P. brasiliensis sensu stricto* S1b | MK909761 |
| HCRP 005 | Lymph node | Subacute | Jaboticabal – SP | *P. brasiliensis* *sensu stricto* S1 | *P. brasiliensis sensu stricto* S1b | MK909762 |
| HCRP 006 | Unknown | Chronic | Ribeirão Preto – SP | *P. brasiliensis* *sensu stricto* S1 | *P. brasiliensis sensu stricto* S1b | MK909763 |
| HCRP 007 | Lymph node | Subacute | Santa Rita de Cássia – MG | *P. brasiliensis* *sensu stricto* S1 | *P. brasiliensis sensu stricto* S1b | MK909764 |
| HCRP 008 | Unknown | Chronic | Monte Santo de Minas – MG | *P. brasiliensis* *sensu stricto* S1 | *P. brasiliensis sensu stricto* S1b | MK909765 |
| HCRP 009 | Lymph node | Chronic | Ibitinga – SP | *P. brasiliensis* *sensu stricto* S1 | *P. brasiliensis sensu stricto* S1b | MK909766 |
| HCRP 054 | Skin biopsy | Chronic | Cravinhos – SP | *P. brasiliensis* *sensu stricto* S1 | *P. brasiliensis sensu stricto* S1a | MK909767 |
| HCRP 055 | Lymph node | Subacute | Barrinha – SP | *P. brasiliensis* *sensu stricto* S1 | *P. brasiliensis sensu stricto* S1b | MK909768 |
| HCRP 056 | Lymph node | Subacute | Ribeirão Preto – SP | *P. brasiliensis* *sensu stricto* S1 | *P. brasiliensis sensu stricto* S1b | MK909769 |
| HCRP 063 | Lymph node | Chronic | Imperatriz – MA | *P. brasiliensis* *sensu stricto* S1 | *P. brasiliensis sensu stricto* S1b | MK909770 |
| HCRP 077 | Mucosa | Subacute | São João Batista da Gloria – MG | *P. brasiliensis* *sensu stricto* S1 | *P. brasiliensis sensu stricto* S1b | MK909771 |
| HCRP 078 | Sputum | Acute | Ribeirão Preto – SP | *P. brasiliensis* *sensu stricto* S1 | *P. brasiliensis sensu stricto* S1b | MK909772 |
| HCRP 080 | Skin biopsy | Subacute | Barrinha – SP | *P. brasiliensis* *sensu stricto* S1 | *P. brasiliensis sensu stricto* S1a | MK909773 |
| HCRP 105 | Skin biopsy | Chronic | Porto Ferreira – SP | *P. brasiliensis* *sensu stricto* S1 | *P. brasiliensis sensu stricto* S1b | MK909774 |
| HCRP 130 | Bronchial alveolar | Chronic | Jaboticabal – SP | *P. brasiliensis* *sensu stricto* S1 | *P. brasiliensis sensu stricto* S1b | MK909775 |
| HCRP 149 | Skin biopsy | Chronic | Morro Agudo – SP | *P. brasiliensis* *sensu stricto* S1 | *P. brasiliensis sensu stricto* S1a | MK909776 |
| HCRP 154 | Skin biopsy | Subacute | Pitangueiras – SP | *P. brasiliensis* *sensu stricto* S1 | *P. brasiliensis sensu stricto* S1b | MK909777 |

**Legend:** MG – Minas Gerais State; MA – Maranhão State; PR – Paraná State; SP – São Paulo State.

**Table S1.** Genotypic characterization of clinical isolates of *Paracoccidioides* spp.: source, clinical PCM form, residence, identification method, and GenBank. (Continued)

|  |  |  |  | ***Identification method*** | |  |
| --- | --- | --- | --- | --- | --- | --- |
| ***Isolate*** | ***Source*** | ***Clinical Form*** | ***Residence*** | ***PCR-RFLP***  ***tub1*** | ***gp43 exon 2 sequencing*** | ***GenBank*** |
| HCRP 157 | Mucosa | Chronic | Batatais – SP | *P. brasiliensis* *sensu stricto* S1 | *P. brasiliensis sensu stricto* S1b | MK909778 |
| HCRP 165 | Mucosa | Chronic | Pradópolis – SP | *P. brasiliensis* *sensu stricto* S1 | *P. brasiliensis sensu stricto* S1b | MK909779 |
| HCRP 168 | Tongue Biopsy | Chronic | Sertãozinho – SP | *P. brasiliensis* *sensu stricto* S1 | *P. brasiliensis sensu stricto* S1b | MK909780 |
| HCRP 170 | Sacral bone | Subacute | Taquaritinga – SP | *P. brasiliensis* *sensu stricto* S1 | *P. brasiliensis sensu stricto* S1b | MK909781 |
| HCRP 171 | Brain Abscess | Chronic | Mococa – SP | *P. brasiliensis* *sensu stricto* S1 | *P. brasiliensis sensu stricto* S1a | MK909782 |
| HCRP 175 | Skin biopsy | Chronic | Caconde – SP | *P. brasiliensis* *sensu stricto* S1 | *P. brasiliensis sensu stricto* S1b | MK909783 |
| HCRP 176 | Bronchial alveolar | Chronic | Ribeirão Preto – SP | *P. brasiliensis* *sensu stricto* S1 | *P. brasiliensis sensu stricto* S1b | MK909784 |
| HCRP 177 | Skin biopsy | Chronic | Ribeirão Preto – SP | *P. brasiliensis* *sensu stricto* S1 | *P. brasiliensis sensu stricto* S1b | MK909785 |
| HCRP 178 | Laryngeal Biopsy | Chronic | Ribeirão Preto – SP | *P. brasiliensis* *sensu stricto* S1 | *P. brasiliensis sensu stricto* S1b | MK909786 |
| HCRP 181 | Tongue Biopsy | Chronic | Santa Cruz das Posses – SP | *P. brasiliensis* *sensu stricto* S1 | *P. brasiliensis sensu stricto* S1b | MK909787 |
| HCRP 183 | Lymph node | Subacute | Serrana – SP | *P. brasiliensis* *sensu stricto* S1 | *P. brasiliensis sensu stricto* S1b | MK909788 |
| HCRP 186 | Lymph node | Subacute | Serra Azul – SP | *P. brasiliensis* *sensu stricto* S1 | *P. brasiliensis sensu stricto* S1b | MK909789 |
| HCRP 187 | Tracheal Biopsy | Chronic | Barrinha – SP | *P. brasiliensis* *sensu stricto* S1 | *P. brasiliensis sensu stricto* S1b | MK909790 |
| HCRP 189 | Mucosa | Chronic | Barrinha – SP | *P. brasiliensis* *sensu stricto* S1 | *P. brasiliensis sensu stricto* S1b | MK909791 |
| HCRP 190 | Bronchial alveolar | Chronic | Ribeirão Preto – SP | *P. brasiliensis* *sensu stricto* S1 | *P. brasiliensis sensu stricto* S1b | MK909792 |
| *HCRP 191 | Skin biopsy | Chronic | Ribeirão Preto – SP | Not identified | *P. lutzii* | MK909806 MK886790 |
| HCRP 192 | Synovial cyst | Subacute | Mococa – SP | *P. brasiliensis* *sensu stricto* S1 | *P. brasiliensis sensu stricto* S1b | MK909793 |
| HCRP 193 | Tracheal Secretion | Chronic | Ituverava – SP | *P. brasiliensis* *sensu stricto* S1 | *P. brasiliensis sensu stricto*S1a | MK909794 |
| HCRP 195 | Mucosa | Chronic | Ribeirão Preto – SP | *P. brasiliensis* *sensu stricto* S1 | *P. brasiliensis sensu stricto* S1b | MK909795 |

**Legend:** MG – Minas Gerais State; MA – Maranhão State; PR – Paraná State; SP – São Paulo State. *HCRP191 had no amplification of the *tub1* gene and was identified by sequencing the *gp43* Exon 2 loci and ITS1/ITS4 rDNA genes.

**Table S1.** Genotypic characterization of clinical isolates of *Paracoccidioides* spp.: source, clinical PCM form, residence, identification method, and GenBank. (Continued)

|  |  |  |  | ***Identification method*** | |  |
| --- | --- | --- | --- | --- | --- | --- |
| ***Isolate*** | ***Source*** | ***Clinical Form*** | ***Residence*** | ***PCR-RFLP***  ***tub1*** | ***Gp43 Exon 2 sequencing*** | ***GenBank*** |
| HCRP 199 | Skin biopsy | Chronic | Ribeirão Preto – SP | *P. americana* (PS2) | *P. americana* (PS2) | MK909796 |
| HCRP 200 | Skin biopsy | Chronic | Ribeirão Preto – SP | *P. brasiliensis* *sensu stricto* S1 | *P. brasiliensis sensu stricto* S1b | MK909797 |
| HCRP FOZ_1 | Sputum | Chronic | Foz do Iguaçu – PR | *P. brasiliensis* *sensu stricto* S1 | *P. brasiliensis sensu stricto* S1b | MK909798 |
| HCRP FOZ_2 | Sputum | Chronic | Foz do Iguaçu – PR | *P. brasiliensis* *sensu stricto* S1 | *P. brasiliensis sensu stricto* S1b | MK909799 |
| HCRP BAT | Lymph node | Subacute | Ribeirão Preto – SP | *P. restrepiensis* (PS3) | *P. restrepiensis* (PS3) | MH484614 |
| HCRP BOAS | Sputum | Chronic | Guatapará – SP | *P. brasiliensis* *sensu stricto* S1 | *P. brasiliensis sensu stricto* S1b | MK909800 |
| HCRP TONI | Sputum | Chronic | Ribeirão Preto – SP | *P. brasiliensis* *sensu stricto* S1 | *P. brasiliensis sensu stricto* S1b | MK909805 |

**Legend:** PR – Paraná State; SP – São Paulo State.
