# Supplementary material for: Phylogenetic Species of Paracoccidioides spp. Isolated from Clinical and Environmental Samples in a Hyperendemic Area of Paracoccidioidomycosis in Southeastern Brazil
Source: J Fungi (Basel). 2020 Aug 11;6(3):132. doi: 10.3390/jof6030132 (PMC7559761; doi:10.3390/jof6030132)
Supplement: Supplementary file 1 [file jof-06-00132-s001.zip › Article_Tiago Cocio_TableS2_22072020.doc]

**Table S2.** Molecular characterization of environmental strains of *Paracoccidioides* spp.: isolate, host, source, geographic origin, identification method, GenBank, and references.

|  |  |  |  |  | ***Identification method*** | |  |  |
| --- | --- | --- | --- | --- | --- | --- | --- | --- |
| ***Isolate*** | ***Host*** | ***Source*** |  | ***Geographic Origin*** | ***PCR-RFLP***  ***tub1*** | ***Gp43 Exon 2 sequencing*** | ***GenBank*** | ***References*** |
| HCRPIBIA | Environment | Soil |  | Ibiá – MG | *P. brasiliensis sensu stricto* S1 | *P. brasiliensis sensu stricto* S1b | MK909801 | [16] |
| HCRPIBIAT1 | Armadillo | Spleen |  |  | *P. brasiliensis sensu stricto* S1 | *P. brasiliensis sensu stricto* S1b | MK909802 | [15] |
| HCRPIBIAT2 | Armadillo | Spleen, Liver, and Lung |  |  | *P. brasiliensis sensu stricto* S1 | *P. brasiliensis sensu stricto* S1b | MK909803 | [15] |
| HCRPIBIAT3 | Armadillo | Spleen and Lung |  |  | *P. brasiliensis sensu stricto* S1 | *P. brasiliensis sensu stricto* S1b | MK909804 | [15] |
