# Supplementary material for: Phylogenetic Species of Paracoccidioides spp. Isolated from Clinical and Environmental Samples in a Hyperendemic Area of Paracoccidioidomycosis in Southeastern Brazil
Source: J Fungi (Basel). 2020 Aug 11;6(3):132. doi: 10.3390/jof6030132 (PMC7559761; doi:10.3390/jof6030132)
Supplement: Supplementary file 1 [file jof-06-00132-s001.zip › Article_Tiago Cocio_TableS3_22072020.doc]

**Table S3.** Reference strains used in the PCR-RFLP techniques of the *tub1* gene, *gp43* Exon 2 loci, and ITS1/ITS4 rDNA: isolate, host, source, geographic origin, identification method, and references.

|  |  |  |  |  | ***Identification method*** | |  |
| --- | --- | --- | --- | --- | --- | --- | --- |
| ***Isolate*** | ***Host*** | ***Source*** | ***Clinical Form*** | ***Residence*** | ***PCR-RFLP***  ***tub1*** | ***Gp43 Exon 2 sequencing*** | ***References*** |
| Pb18 or B17 | Human | Unknown | Chronic | São Paulo – SP | *P. brasiliensis sensu stricto* S1 | *P. brasiliensis sensu stricto* S1b | [5] |
| EPM194 or Pbdog | Dog | Lymph node | - | Curitiba - PR | *P. americana* (PS2) | *P. americana* (PS2) | [17] |
| EPM54 or T2 | Environment | Soil | - | Caracas, Venezuela | *P. restrepiensis* (PS3) | *P. restrepiensis* (PS3) | [18] |
| Pb01 | Human | - | Chronic | Goiania - GO | *P. lutzii* | *P. lutzii* | [4] |

**Legend:** GO – Goiás State; PR – Paraná State; SP – São Paulo State.
