# Supplementary material for: Phylogenetic Species of Paracoccidioides spp. Isolated from Clinical and Environmental Samples in a Hyperendemic Area of Paracoccidioidomycosis in Southeastern Brazil
Source: J Fungi (Basel). 2020 Aug 11;6(3):132. doi: 10.3390/jof6030132 (PMC7559761; doi:10.3390/jof6030132)
Supplement: Supplementary file 1 [file jof-06-00132-s001.zip › Article_Tiago Cocio_TableS4_22072020.doc]

**Table S4.** Specific primers, size of PCR products, annealing temperatures, and references of the used methods.

| ***Gene*** | ***Primer*** | **Sequence (5'->3')** | **Annealing temperature (°C)** | ***Amplicon***  **(bp)** | ***Reference*** |
| --- | --- | --- | --- | --- | --- |
| *tub*1 | *tub1F* | CTG GGA GGT ATG ATA ACA CTG C | **60** | **263** | [18] |
|  | *tub1R* | CGT CGG GCT ATT CAG ATT TAA G |  |  |  |
| *gp43* exon 2 loci | *Pbgp43-E2F* | CTA GAA TAT CTC ACT CCC AG | **58** | **722** | [10] |
|  | *Pb_gp43-E2R* | GCC CCC TCC GTC TTC CAT GTC C |  |  |  |
| ITS1-5.8S-ITS2 | *ITS 1* | TCC GTA GGT GAA CCT GCG G | **65** | **633** | [10, 21] |
|  | *ITS 4* | TCC TCC GCT TAT TGA TAT GC |  |  |  |

ITS, internal transcribed spacer.
